# The effect of clay type on the toxicity of carbendazim and imidacloprid to the earthworm *Eisenia andrei* in artificial soils

**DOI:** 10.1007/s10646-025-02889-6

**Published:** 2025-04-26

**Authors:** Bart G. van Hall, Shuan Meijer, Anna C. Pelser, Cornelis A. M. van Gestel

**Affiliations:** Amsterdam Institute for Life and Environment (A-LIFE), Faculty of Science, Vrije Universiteit Amsterdam, HZ Amsterdam, The Netherlands

**Keywords:** Bentonite clay, Kaolin clay, Soil risk assessment, Pesticides, Soil invertebrates

## Abstract

In Europe, the risk assessment of pesticides to soils organisms is based on standardized laboratory toxicity tests using artificial soil containing kaolin clay. However, kaolin is not the most representative clay type for European agricultural soils, and its use may affect the bioavailability and toxicity of pesticides, potentially leading to an underestimation of the actual risk to soil organisms. In this study, reproduction toxicity tests with the earthworm *Eisenia andrei* following OECD guideline 222 were performed in artificial soils prepared with kaolin or bentonite clay, using the pesticides carbendazim and imidacloprid. The results showed that the OECD guideline 222 quality criteria could be met in soils prepared with bentonite clay. EC_50_ reproduction values (and 95% CIs) in soils prepared with kaolin and bentonite clay were 1.80 (1.02–2.57) and 4.19 (−10.4–18.8) mg kg^−1^ for carbendazim, and 0.71 (0.06–1.36) and 2.27 (−0.26–4.80) mg kg^−1^ for imidacloprid. For both pesticides, toxicity (LCx, ECx biomass, ECx reproduction) was higher in soils prepared with kaolin clay, although the differences were not always statistically significant. Differences in toxicity between the soils were likely due to a combination of the bentonite’s larger interlayer distance, providing space for the pesticides to enter in between the clay sheets, and the higher cation exchange capacity (7.30 and 22.8 cmol_c_ kg^−1^ for kaolin and bentonite soil, respectively) leading to increased pesticide sorption. Overall, these findings suggest that kaolin is a suitable clay type for standardized artificial soil, as it exhibited the highest toxicity, and thus provided a “worst-case” scenario.

## Introduction

The European Food Safety Authority (EFSA) evaluates the potential risks associated with pesticides to human and environmental health. The risks to soil invertebrates are established with the help of standardized laboratory toxicity tests conducted with, among others, *Eisenia andrei* or *Eisenia fetida*, which are considered representative earthworm species. These toxicity tests are usually performed according to the guideline for the testing of chemicals number 222 of the Organization for Economic Co-operation and development (OECD), using an artificial test soil made up of quartz sand, kaolin clay, sphagnum peat, and some CaCO_3_ to adjust pH (OECD, 2016).

Kaolinite is a widespread 1:1 clay mineral (Al_2_Si_2_O_5_(OH)_4_), in which a clay layer consists of a tetrahedral silica sheet and an octahedral alumina sheet (Shainberg and Levy, 2005). Kaolinite has a low negative surface charge on the clay particle surface (Murray, 2006). It has a small interlayer distance of approximately 0.70 nm, hindering water molecule entry, and resulting in a low Cation-Exchange Capacity (CEC) of <10 cmol_c_ kg^−1^ because ions are not able to exchange efficiently (Kumari and Mohan, 2021; Zhang et al. 2022). Due to these properties, the sorption capacity of kaolinite is low (Murray, 2006), which is why it was preferred in the original development of artificial soils over other readily available clay types such as bentonite (Edwards, 1984).

Although kaolinite clay is widely abundant, it is not necessarily the most representative clay type for European agricultural soils, as they also contain 2:1 clay minerals such as montmorillonite and illite. These 2:1 clay minerals have higher sorption capacities due to the high surface area and high layer charge (Murray, 2006; Kumari and Mohan, 2021). Bentonite (Al_2_O_3_(4(SiO_2_)H_2_O) is composed of 98% montmorillonite, and its clay layers consist of an octahedral alumina sheet in between two tetrahedral silica sheets (Shainberg and Levy, 2005; Kumari and Mohan, 2021). It has a larger interlayer distance than kaolinite, of 0.96 to 2.0 nm, which is due to the slight attraction between oxygen atoms between the sheets (Jiang, 2021; Kumari and Mohan, 2021). This increased space between layers allows for water retention, and goes along with a higher CEC ranging from 80 to 100 cmol_c_ kg^−1^ (Lehmann et al. 2021; Murray, 2006).

The difference in sorption capacities between clay types may influence the bioavailability and toxicity of pesticides to earthworms, as they influence the porewater concentration which is considered their primary route of exposure to chemicals in soil (Van Hall et al. 2024). This may be especially relevant for dissociating pesticides such as carbendazim, or polar pesticides such as imidacloprid, as the pesticide molecules are electrically charged and can adsorb to the negatively charged clay surface, reducing the porewater concentration. Indeed, several studies have previously reported higher imidacloprid toxicity to earthworms and springtails in natural LUFA 2.2 soil than in artificial soil, which was often attributed to the different clay contents and types (Ogungbemi and van Gestel, 2018; Bandeira et al. 2020; Van Hall et al. 2024). This suggests that the use of kaolin clay may lead to a lower bioavailability of certain pesticides in toxicity tests, potentially underestimating the risk posed in natural soils, which contain different clay types.

Therefore, the goal of this study was to evaluate the potential use of bentonite clay as a substitution for kaolin clay in artificial soils, and to investigate whether the use of kaolin clay provides a “worst-case” scenario in terms of pesticide toxicity to earthworms. The focus was on answering two questions: (1) Does clay type influence the performance of *E. andrei* in artificial soils? And (2) Does clay type influence the toxicity of carbendazim and imidacloprid to *E. andrei*?

## Material and methods

### Test organism

Earthworms (*Eisenia andrei*) were cultured at the Amsterdam Institute for Life and Environment (A-LIFE), Section Ecology & Evolution, Vrije Universiteit Amsterdam, The Netherlands. The culture was kept in complete darkness at 20 ± 2 °C with 75% relative humidity on a 1:1 (v:v) substrate of potting soil and sphagnum peat. The substrate was moistened with tap water to approximately 50% of the maximum water holding capacity (WHC_max_), and CaCO_3_ was added to bring the pH (0.01 M CaCl_2_) to 6.0 ± 1.0. The earthworms were fed weekly with manure from healthy horses obtained from a local horse-riding school that were not treated with any medication in the previous six weeks. Earthworms were age-synchronized by allowing adults to lay cocoons in fresh substrate over a period of four weeks, after which they were removed. After an additional 16 weeks, the earthworms had developed a clitellum and were ready to be used in the toxicity tests.

### Test soils

Two artificial soils were prepared based on the OECD guideline 222 (OECD, 2016), but using different types of clay. The artificial soils consisted of 10% dried, finely ground (<2 mm) sphagnum peat (Jongkind, The Netherlands), 20% kaolin or bentonite clay (Keramikos, The Netherlands), 70% quartz sand with a grain size of 0.1–0.3 mm (Multiquartz, The Netherlands) and CaCO_3_ (Merck, Germany) to adjust the soil pH (0.01 M CaCl_2_) to 6.0 ± 0.5. The amount of CaCO_3_ needed to set the soil pH to 6.0 ± 0.5 was determined by constructing pH-calibration curves (see Fig. S1 in the Supplementary Information). Dry constituents were mixed well and stored at 20 ± 2 °C until further use. Soil WHC_max_ was measured as described in Annex 2 of OECD guideline 222 (OECD, 2016). Six days before the toxicity tests started, the artificial soils were moistened to 25% of their respective WHC_max_ to equilibrate the acidity levels. Soil pH was measured at the start and end of the experiments by shaking 6 g portions of the moist soil with 24 mL 0.01 M CaCl_2_ for two hours at 200 rpm. The pH of the suspensions was measured after settling overnight. Soil organic matter content was measured as loss on ignition by first drying moist soil samples (5 g) at 100 °C, and then ashing the samples at 200 and 400 °C for 1 h, and at 500 °C for 6 h. Cation exchange capacity of the soils was measured by Normec Groen Agro Control (Delfgauw, The Netherlands) using the BaCl_2_ single-extract method at soil pH (Nel et al. 2023). An overview of the soil properties is shown in Table 1.

**Table 1:** Properties of OECD artificial soils prepared with kaolin or bentonite clay, used to determine the toxicity of carbendazim and imidacloprid to the earthworm *Eisenia andrei*

Soil properties	Kaolin	Bentonite
Sphagnum peat (%)	10.0	10.0
Organic matter (%)^a^	9.87 ± 0.67	10.1 ± 0.14
Clay content (%)	20.0	20.0
Quartz sand (%)	70.0	70.0
pH (0.01 M CaCl_2_)^b^	5.41 ± 0.07	5.77 ± 0.03
WHC_max_ (%)^c^	55.2 ± 0.56	73.8 ± 1.31
Cation Exchange Capacity (cmol_c_ kg^−1^)	7.30	22.8

^a^Average ± SD of lowest test concentrations (n = 4)

^b^Average ± SD of (solvent) controls at the start of the tests (n = 6)

^c^Average ± SD of artificial soil containing kaolin (n = 6) or bentonite (n = 4)

### Soil spiking and chemical analysis

An overview of the suppliers, purity and chemical properties of imidacloprid and carbendazim are shown in Table 2. Nominal test concentrations were based on literature data (Van Hall et al. 2023). For imidacloprid, these were 0.26, 0.64, 1.6, 4, 10, and 25 mg kg^−1^ dry soil and 0.64, 1.6, 4, 10, 25 and 62.5 mg kg^−1^ dry soil for the kaolin and bentonite soils, respectively. Imidacloprid was dissolved in demineralised water and mixed in with the pre-moistened soils to achieve the desired pesticide concentrations for each treatment, as well as a moisture content of 50% WHC_max_. For carbendazim, nominal test concentrations were 0.67, 2.0, 6.0, 18, and 54 mg kg^−1^ dry soil for both soils. Carbendazim was dissolved in acetone and the required amount of pesticide solution was added to 10% (w/w) of the amount of soil required for each test concentration in such a way that approximately 1 mm of acetone was standing on top of the soil. After equilibration for 6 h, the glass jar (800 mL) containing the spiked soil was opened in a fume hood to allow overnight evaporation of the acetone. The following day, the spiked soil was mixed with the remaining 90% of the pre-moistened soil, and demineralized water was added to reach 50% WHC_max_. All soils were mixed thoroughly to ensure that the test substance was evenly distributed throughout the soil. For each treatment, the soil was divided over four replicates (500 g moist soil per replicate; 800 mL glass test jars). Controls were included for both substances, and an acetone solvent control was added for carbendazim. Accuracy of the soil spiking procedure was later confirmed through chemical analysis, and is described in a subsequent section.

**Table 2:** Suppliers, purity and chemical properties of the pesticides used in the earthworm toxicity tests in artificial soils with different clay types^a^

	Carbendazim	Imidacloprid
Manufacturer	Sigma Aldrich	Bayer Crop Science
Purity (%)	97.0	98.0
Log K_ow_	1.5	0.57
pK_a_	4.2	-
Water solubility (mg L^−1^)	8.0	610

^a^Data on chemical properties are from the Pesticide Properties Database (Lewis et al. 2016)

### Toxicity tests

Toxicity tests followed OECD guideline 222 (OECD, 2016) and were conducted in the same week for each pesticide with earthworms originating from the same culture box. Test jars were kept in a climate room set at 20 °C, with 75% relative humidity and a 12:12 h light-dark photoperiod. One day before the test, age-synchronised clitellate adults were acclimatised in the corresponding uncontaminated soil moistened to 50% WHC_max_. The following day, ten earthworms were randomly selected, rinsed with water, and blotted dry with tissue paper. Three randomly selected worms from each group were weighed individually to assess variation in starting weights, while the remaining seven worms were weighed as a group. Mean individual earthworm weights (based on the three individually weighed earthworms for each replicate) for soils containing kaolin and bentonite clay ( ± SD; n = 84) were 432 ± 47 and 437 ± 23 mg for the tests with imidacloprid, and 449 ± 81 and 437 ± 63 mg for the carbendazim tests, respectively. After weighing, the earthworms were placed on the soil surface in the test jars of a randomly assigned replicate of a concentration (n = 4) or (solvent) control (n = 4). During the first four weeks, jars were weighed once a week, demineralized water was added to account for evaporation, and 7 grams of food was provided per replicate. The food consisted of a mixture of finely ground horse manure and ground oats moistened with demineralised water in a ratio of 1:5 (w/v). To prevent mould formation, the food mixture was inserted into a hole in the soil and subsequently covered with soil.

After 28 days of exposure, the adult worms were removed by hand-sorting, counted, rinsed with water, blotted dry with tissue paper, and weighed. The remaining soil with the produced cocoons was returned into the test jars and left to incubate for an additional four weeks under the same test conditions, with the exception that food was given only once at the beginning of the four week incubation period. After four weeks, the test jars were placed in a 55 °C water bath for 30 min and all juveniles emerging to the soil surface were collected and counted. Following the water bath incubation, soils were manually searched to extract any remaining juveniles. To assess the validity of the tests, the results obtained in the controls were compared with the validity criteria specified in the OECD guideline 222 (OECD, 2016).

### Chemical analysis

At the start and end of the tests, soil samples (approximately 20 g; n = 2) were collected for each test concentration and subsequently kept at −20 °C. The soils were used to quantify actual pesticide concentrations at the start of the tests in the solvent control for carbendazim, the control for imidacloprid, and for both compounds in soils spiked with the concentrations surrounding the EC_50_ values for effects on reproduction. Pesticide extractions were done using exhaustive measures and the limit of detection was 0.01 mg kg^−1^ soil. Measurements were performed by Normec Groen Agro Control (Delfgauw, The Netherlands) applying the QuEChERs method (Anastassiades et al. 2003) and using LC-MS/MS, following certified chemical analytical procedures and quality control measures. Quality control included the measurement of independent control standards at the start and end of the measurement sequence to ensure accurate calibration and instrument performance. Soil samples were spiked with known quantities of carbendazim and imidacloprid to verify extraction efficiency and determine recovery rates. The recovery percentages ranged between 70 and 120%, with corrections applied if values fell outside these criteria. All samples were processed by the same individual and analysed using the same instrument to minimize variability. Since the pesticide concentrations were quantified in moist soils at 50% WHC_max_, the measured values were corrected for moisture content to give mg kg^−1^ dry soil values before data analysis. Soil collected at the end of the test was used to measure soil pH as previously described.

### Data analysis

Statistical analyses were performed in RStudio (version 2023.12.0) running R version 4.2.1 (R Core Team, 2021), and in Microsoft Excel (version 2406). The average individual biomass of earthworms was calculated for each test jar by dividing the total earthworm biomass by the number of surviving adults. Homogeneity of variance for earthworm survival, average individual biomass, and reproduction in the control and solvent control jars were compared by performing Levene’s tests. Depending on the outcome, unpaired Student’s t-tests assuming equal or unequal variances were performed. When no significant differences between control and solvent control groups were detected, the groups were combined for the subsequent data analysis.

For each pesticide, earthworm performance (i.e. survival, average individual biomass, and reproduction) in the (solvent) control of the two soils were compared by conducting Levene’s tests followed by unpaired Student’s t-tests. The dose-response data obtained from the toxicity tests were used to fit three-parameter log-logistic models in RStudio using the “*drc*” package (Ritz et al. 2015), from which the toxicity values (LC_50_, LC_10_, EC_50_ and EC_10_ for effects on survival, reproduction and final individual biomass) and corresponding 95% confidence intervals were extracted. Since biomass changes showed an increase at low and a decrease at high concentrations, this complicated the fitting of dose-response relationships. We therefore assumed a homogenous distribution of earthworm masses at the start of the tests, and focussed our analysis on final individual earthworm masses at the end of the exposures. To assess the significance of differences in pesticide toxicity between the soils, generalized likelihood ratio tests were performed comparing the dose-response models to a constrained version where similar toxicity in the soils was assumed (i.e. same LC_50_, LC_10_, EC_50_ or EC_10_ values; Zhang and Van Gestel, 2019).

## Results

### Soil properties and chemical measurements

Soil pH in the controls at the start of the tests was approximately 0.6 pH units lower than expected for the soil containing kaolin clay, and 0.25 pH units lower for the soil containing bentonite clay (Table 1). Pesticide addition to the soils did not influence soil pH, and soil pH in the (solvent) controls remained constant (±0.25 pH units) during the tests (Table S1). The CEC differed between the soils by approximately a factor of 3, with CEC values of 7.3 cmol_c_ kg^−1^ and 22.8 cmol_c_ kg^−1^ for soils prepared with kaolin and bentonite clay, respectively.

No pesticides were measured in the solvent control soils. For carbendazim, the measured concentrations (±SD; n = 2) in kaolin and bentonite soils were 104 ± 3.23 and 94.8 ± 1.61%, respectively of the nominal concentrations. For imidacloprid, the measured concentrations (±SD; n = 2) in kaolin and bentonite soils were 85.7 ± 8.45 and 95.9 ± 14.5%, respectively of the nominal concentrations. As the measured concentrations were within the acceptable range of 70–120% of the nominal concentrations (EC, 2021) and did not differ much between soils, data analyses were performed with nominal concentrations to avoid adding additional complexity and uncertainty which might occur when correcting for the measured values.

### Validity of the toxicity tests

An overview of earthworm performance in the control jars of the toxicity tests is shown in Table 3. For the tests with imidacloprid, the validity criteria were met in both soils. For the tests with carbendazim, the validity criteria for reproduction were not met as the minimum number of juveniles produced per test jar was 25 and 18 for kaolin and bentonite soils, respectively, and the coefficient of variation was slightly too high at 32.0% in the kaolin soil. However, as the average number of juveniles in the kaolin soil was 37.8 per control jar, and the data showed consistent dose-related responses to carbendazim in both soils, this was not considered problematic for the aims of the current study.

**Table 3:** Control performance (±SD; n = 4) of *Eisenia andrei* in earthworm toxicity tests using OECD artificial soils prepared with kaolin or bentonite clay

Pesticide	Clay type	Average (± SD) adult survival (%)	Minimum # of juveniles per test jar	Average (± SD) # of juveniles per test jar	Coefficient of Variation (%) of juvenile numbers
Carbendazim	Kaolin	97.5 ± 0.50	25	37.8 ± 12.1	32.0
	Bentonite	97.5 ± 0.50	18	22.8 ± 4.11	18.1
Imidacloprid	Kaolin	95.0 ± 5.8	43	54.5 ± 8.19	15.0
	Bentonite	100 ± 0.00	35	41.0 ± 6.06	14.8
**Validity criteria**		**≥90**	**≥30**	**-**	**≤30**

### Earthworm performance in two artificial soils

**Fig. 1:**
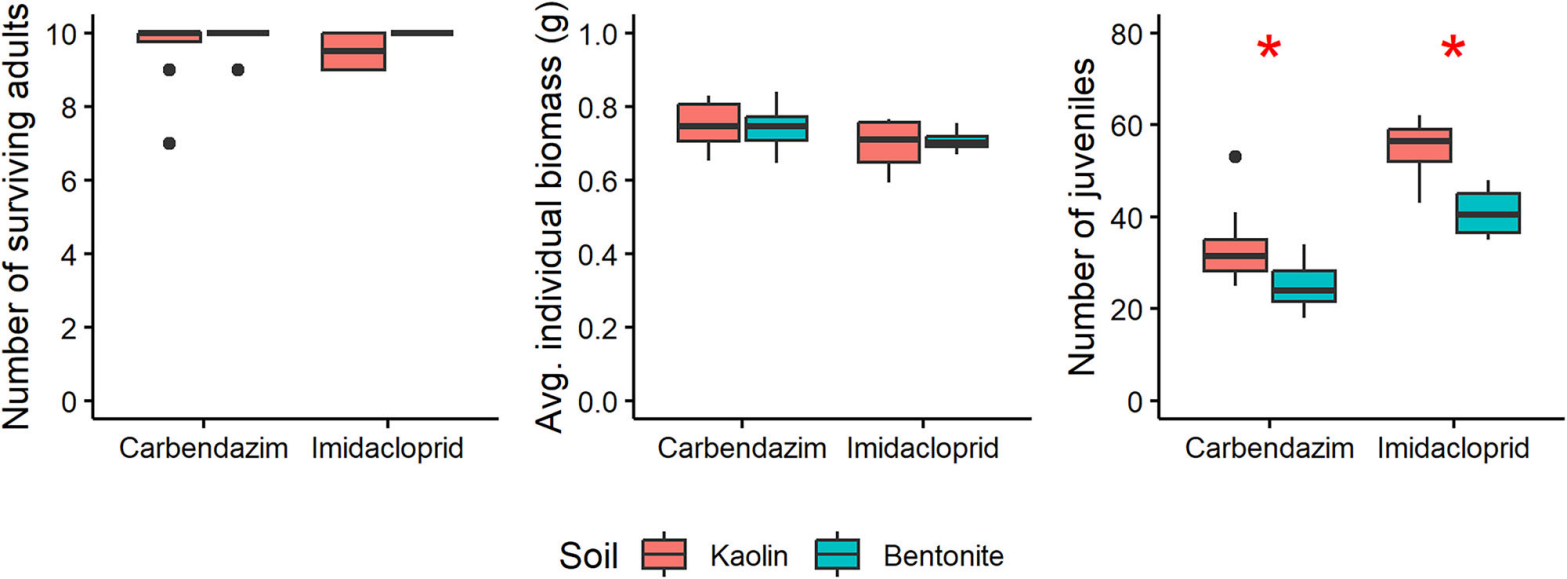
Boxplots of adult survival (left) and average individual biomass (middle) after 28 days, and number of juveniles produced (right) after 56 days by the earthworm *Eisenia andrei* in (solvent) controls of toxicity tests with carbendazim (n = 8) and imidacloprid (n = 4) in artificial soils constructed with kaolin and bentonite clay. The middle line of each box represents the median. The top and bottom edges of the box represent the 75th and 25th percentiles, respectively. Whiskers extend to the smallest and largest values within 1.5 times the interquartile range. Data points beyond this range are shown as dots and represent outliers. * denotes significant differences at p < 0.05 (Student’s t test)

For carbendazim, the data for the controls and solvent controls were pooled together for each soil, as there were no significant differences in earthworm survival, biomass change, and reproduction (Student’s t test, p > 0.05). Earthworm biomass (mean ± SD; n = 8) increased by 66.1 ± 15.7 and 69.4 ± 9.42% in the (solvent) controls of carbendazim and by 54.1 ± 21.2 and 64.1 ± 5.77% in the controls (n = 4) of imidacloprid in the kaolin and bentonite soils, respectively (Fig. S2). For both pesticides, no significant differences were observed in earthworm survival and final individual biomass in control soils containing kaolin and bentonite clay (Student’s t test, p > 0.05; Fig. 1). In contrast, the number of juveniles produced was significantly lower in the soils containing bentonite clay, for both carbendazim (Student’s t test, p = 0.03) and imidacloprid (Student’s t test, p = 0.04), indicating that earthworm performance was better in the standard artificial soil constructed with kaolin clay.

### Pesticide toxicity

**Fig. 2:**
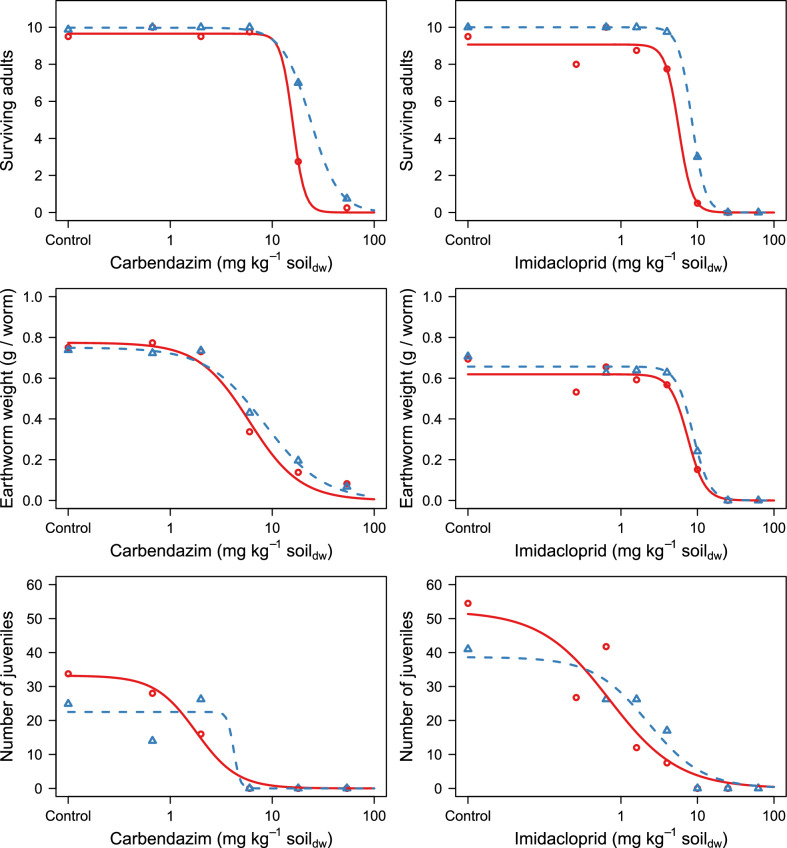
Dose-response curves for the effects of carbendazim (left) and imidacloprid (right) on the survival (top), average individual biomass (middle) and reproduction (bottom) of *Eisenia andrei* exposed in artificial soils containing kaolin (red) and bentonite (blue) clays. Points indicate average values (n = 4; n = 8 for carbendazim controls) and lines depict the fit of a three-parameter logistic dose-response model

For both carbendazim and imidacloprid in artificial soils containing kaolin and bentonite clays, earthworm survival, individual biomass and reproduction showed a dose-dependent decrease (Fig. 2). Table 4 displays the toxicity values (LC_x_ and EC_x_) derived from these dose-response relationships.

**Table 4:** Toxicity values (LC_x_ and EC_x_; ±95% confidence intervals) for the effects of carbendazim and imidacloprid on the survival, average individual biomass, and reproduction of the earthworm *Eisenia andrei* in artificial soils prepared with kaolin and bentonite clays

	Carbendazim (mg kg^−1^ dry soil)			Imidacloprid (mg kg^−1^ dry soil)		
Endpoint	Kaolin	Bentonite	Ratio	Kaolin	Bentonite	Ratio
LC_50_	15.9*** (6.70–25.1)	23.8*** (20.3–27.2)	1.50	5.69* (4.48–6.91)	8.43* (6.57–10.3)	1.48
LC_10_	11.9 (−11.3–35.0)	11.7 (8.96–14.5)	0.98	3.67 (2.81–4.52)	5.42 (1.61–9.23)	1.48
EC_50_ – Biomass	5.99* (4.71–7.27)	8.45* (6.42–10.5)	1.41	7.45 (5.91–8.98)	8.72 (7.12–10.3)	1.17
EC_10_ – Biomass	1.69 (0.91–2.47)	1.97 (1.00–2.94)	1.17	4.20 (2.25–6.15)	5.06 (1.85–8.27)	1.20
EC_50_ - Reproduction	1.80 (1.02–2.57)	4.19 (−10.4–18.8)	2.33	0.71 (0.06–1.36)	2.27 (−0.26–4.80)	3.20
EC_10_ – Reproduction	0.64*** (−0.07–1.36)	3.60*** (−12.6–19.8)	5.63	0.07 (−0.08–0.23)	0.37 (−0.58–1.31)	5.29

Ratios between toxicity values obtained in soils constructed with kaolin and bentonite clay are also provided. All toxicity values are based on nominal pesticide concentrations

^*^indicates significant differences in toxicity at p < 0.05

^***^indicates significant differences in toxicity at p < 0.001

For carbendazim, LC_50_ values were significantly lower in kaolin soil than in bentonite soil (χ^2^_df=1_ = 22.8; p < 0.001), differing by a factor of 1.50 (Table 4), but no significant differences were seen between LC_10_ values (χ^2^_df=1_ = 0.01; p > 0.05). Earthworm biomass change showed dose-related decreases upon carbendazim exposure in both kaolin and bentonite soils (Fig. S2). Effects on earthworm individual final biomasses showed significant differences between carbendazim EC_50_ values (χ^2^_df=1_ = 4.88; p = 0.03), but not between EC_10_ values (χ^2^_df=1_ = 0.22; p > 0.05). For earthworm reproduction, the EC_50_ was a factor of 2.33 lower in kaolin soil than in bentonite soil, but the difference was not significant (χ^2^_df=1_ = 0.64; p > 0.05). In contrast, the EC_10_ values were significantly different between soils (X^2^_df=1_ = 195.5, p < 0.001), with values of 0.64 mg kg^−1^ dry soil and 3.60 mg kg^−1^ dry soil for kaolin and bentonite soils, respectively.

Imidacloprid was also most toxic in the kaolin soil compared to the bentonite soil (Table 4). The generalized likelihood ratio test showed a significant difference in LC_50_s (X^2^_df=1_ = 5.17; p = 0.02), with values differing by a factor of 1.48 (Table 4), but not in LC_10_ values (X^2^_df=1_ = 2.29; p > 0.05). Earthworm biomass was dose-related decreased by imidacloprid in both kaolin and bentonite soils spiked with imidacloprid (Fig. S2). The EC_50_ and EC_10_ values for individual biomass were in a similar range, and no significant differences were seen between earthworm biomass EC_50_ (X^2^_df=1_ = 1.32; p > 0.05) and EC_10_ (X^2^_df=1_ = 0.27; p > 0.05) values. The EC_50_ and EC_10_ values for reproduction differed by factors of 3.20 and 5.29 between the soils, but were not significantly different (X^2^_df=1_ = 2.86 and X^2^_df=1_ = 1.04, respectively; p > 0.05).

## Discussion

### Effect of clay type on earthworm performance

Assessing earthworm performance is crucial to assess whether soils are potentially suitable for use in ecotoxicological tests and environmental risk assessment (ERA) studies. In this study, earthworms produced significantly more juveniles in the artificial soil prepared with kaolin than with bentonite clay, while the survival and average individual biomass in the (solvent) controls of both soils did not differ significantly (Fig. 1). Despite the lower number of juveniles produced in soils containing bentonite, the validity criteria were still met for the tests performed with imidacloprid (Table 3), indicating that substituting kaolin clay with bentonite clay can be performed successfully, and that validity criteria do not need to be redefined.

### Comparing toxicity values with literature data

Van Hall et al. (2024) reported a 28-day LC_50_ of 14 mg kg^−1^ dry soil and a 56-day EC_50_ of 2.0 mg kg^−1^ dry soil (nominal concentrations) for the toxicity of carbendazim to *E. andrei* exposed in artificial soil containing kaolin clay, which aligns well with the values (based on nominal concentrations) of 15.9 mg kg^−1^ dry soil and 1.80 mg kg^−1^ dry soil found in this study. The toxicity values were a factor of 2–5 higher than values found by De Silva et al. (2009) who reported an LC_50_ of 7.1 mg kg^−1^ and an EC_50_ of 0.39 mg kg^−1^, and Robidoux et al. (2004) who found an EC_50_ of 0.88 mg kg^−1^ in artificial soil. Ellis et al. (2007) exposed *Eisenia fetida* to carbendazim in artificial soil constructed with bentonite clay and reported a 14-day LC_50_ of 8.03 mg kg^−1^, which is a factor of 3 lower than the value of 23.8 mg kg^−1^ found in this study_._

Van Hall et al. (2024) also reported LC_50_ and EC_50_ values (based on nominal concentrations) for imidacloprid in artificial soil of 9.6 mg kg^−1^ dry soil and 3.4 mg kg^−1^ dry soil, respectively, which are somewhat higher than the values of 5.7 mg kg^−1^ dry soil and 0.7 mg kg^−1^ dry soil obtained in this study. However, Wang et al. (2019) reported an EC_50_ of 0.87 mg kg^−1^ dry soil, which is in good agreement with our results.

Overall, the toxicity data found in this study are well in agreement with earlier findings, as differences were within a factor of 5, which is the standard range of interlaboratory variation (Fairbrother, 2008). Thus, the toxicity tests were adequately performed and the results are reliable.

### Effect of clay type on carbendazim and imidacloprid toxicity

The effect of clay type on pesticide toxicity was very similar for carbendazim and imidacloprid, as indicated by the high similarity in toxicity ratios displayed in Table 4. For both pesticides, the toxicity was higher in soils containing kaolin clay than in soils containing bentonite clay, and the largest differences in toxicity were seen for the reproduction endpoints. For carbendazim significant differences in LC_50_, EC_50_ biomass, and EC_10_ reproduction were observed, while only LC_50_ values significantly differed for imidacloprid (Table 4).

To understand what factors drive the observed differences in toxicity between soils, it is important to understand in what chemical form the pesticides are present. This is especially important for carbendazim, as it has a pKa of 4.2 (Table 2) and can therefore be in a neutral or protonated form which determines interactions with soil components. The degrees of protonation of carbendazim were calculated using the Henderson-Hasselbach equation:

$${\rm{Fraction}}\,{\rm{protonated}}=\frac{1}{(1+{10}^{pKa-pH})}$$

The degrees of protonation for carbendazim were low in both soils, being 7% in the kaolin soil (pH at t = 0 was 5.43) and 3% in the bentonite soil (pH at t = 0 was 5.77). In both soils, carbendazim was thus mostly present in the neutral form.

**Fig. 3:**
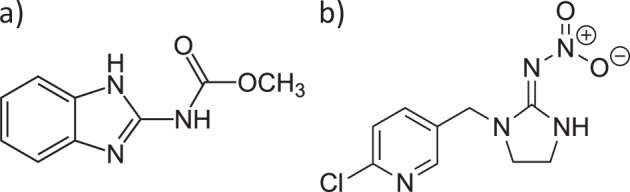
2-D chemical structures of carbendazim (**a**) and imidacloprid (**b**)

Most likely, the differences in toxicity are caused by the factor of three difference in CEC between the soils, as the other soil properties were similar (Table 1). The difference in CEC is due to bentonite clay having silicates with expanding layers and therefore both internal and external surfaces being the active sites and having a great propensity for cation exchange and adsorption, while kaolinite has no expanding layers and only has an external surface as an adsorption site (Yu et al. 2013). Both carbendazim and imidacloprid can be considered polar compounds. Carbendazim contains amine (–NH) and carbamate (–NHCOO) functional groups (Fig. 3a), while imidacloprid contains a nitro (–NO_2_) group (Fig. 3b). As a results, both compounds may interact with the charged surface of the clay particles. If this is the case, sorption to the clay surface will reduce the pesticide concentration in the pore water, which is considered the primary route of chemical exposure for soil invertebrates (Styrishave et al. 2010), thus leading to lower toxicity. However, to access the internal cation exchange sites in bentonite clay, the pesticide molecules need to be able to fit in between the clay sheets.

To verify this hypothesis, the 3D structures of carbendazim and imidacloprid were obtained from the PubChem database (Kim et al. 2023), and the molecular visualization tool PyMOL version 3.0.4 (DeLano, 2002) was used to visualize the molecules and to measure their dimensions. For carbendazim, the x, y and z measurements were 1.12, 0.50, and 0.18 nm, respectively, and for imidacloprid 0.91, 0.60, and 0.47 nm, respectively. As the interlayer distance of bentonite is between 0.96 to 2.0 nm (Kumari and Mohan, 2021; Jiang, 2021), both pesticide molecules should indeed fit between the bentonite layers, accessing the internal cation exchange sites. In contrast, the interlayer distance for kaolinite sheets is only approximately 0.70 nm (Kumari and Mohan, 2021; Zhang et al. 2022), meaning that carbendazim and imidacloprid cannot fit between the sheets of this clay type.

Similar findings have been reported for the herbicide paraquat, which also has nitrogen atoms carrying a positive charge, thereby facilitating sorption to negatively charged clay surfaces. For instance, Tucker et al. (1969) mentioned that paraquat strongly sorbed to bentonite and montmorillonite, and that sorption to kaolinite was less effective. Furthermore, Ye and Lemley (2008) studied the adsorption of paraquat, carbaryl and mecoprop to montmorillonite clay and found that, unlike carbaryl and mecoprop, paraquat strongly sorbs to clay and positions itself within the clay sheets. In fact, paraquat sorption within the clay matrix was so strong that no effective method to extract it could be established, which was likely caused by the flatness of the molecule. As carbendazim molecules are also flat and contain amine- and carbamate functional groups, it is likely that sorption within a clay matrix is also very strong. Imidacloprid on the other hand, is not a flat molecule, and sorption within the clay matrix therefore is less likely. These assumptions, however, require further investigation.

The lower imidacloprid toxicity in soils containing bentonite clay compared to kaolin clay was somewhat surprising, as other papers mentioned higher imidacloprid toxicity in natural LUFA 2.2 soil than in artificial soils containing kaolin clay (Ogungbemi and van Gestel, 2018; Bandeira et al. 2020), even when the organic matter content is similar between the soils (Van Hall et al. 2024). These differences are usually attributed to differences in clay content, as LUFA 2.2 contains approximately 11% clay compared to the 20% clay used in artificial soil, and to clay type, as LUFA 2.2 contains 2:1 clay minerals as opposed to the 1:1 kaolinite clay minerals. While this may be true, it is expected that these differences would be reflected in the respective CECs of the soils, which is not the case, as according to LUFA Speyer (Speyer, Germany) the CEC of LUFA 2.2 soil is 9.54 cmol_c_ kg^−1^ which is similar to the CEC of 7.30 cmol_c_ kg^−1^ in artificial soil containing kaolin clay found in the current study. Consequently, the degree of sorption caused by the polar group of imidacloprid would be expected to be similar between the soils. Therefore, it seems likely that the differences in imidacloprid toxicity are caused by differences in organic matter type (i.e. naturally weathered organic matter versus sphagnum peat) leading to differences in sorption of the non-polar hydrophobic part of the molecule (Hofman et al. 2008; Bielská et al. 2014).

### Implications for risk assessment

The results from this study show that kaolin clay, which is currently used in standardized artificial soils, generally provides a “worst-case” scenario for pesticide toxicity compared to the more environmentally relevant bentonite clay. This ensures that the risks to non-target organisms, such as earthworms, are not underestimated in the environmental risk assessment. From a regulatory perspective, this aligns with the precautionary principle, as the use of kaolin minimizes the likelihood of overlooking potential hazards.

However, the reduced environmental relevancy of the kaolin test soil could also potentially cause ERAs to be too conservative. To enhance the environmental relevance of standardized toxicity tests, regulatory frameworks could consider supplementary testing with alternative soil types, such as artificial soil containing bentonite clay or even natural soils as an intermediate tier (Ernst et al. 2024), to account for variability in soil properties. This dual approach could improve the balance between protective measures and environmental realism in ERAs.

## Conclusion

This study showed that earthworm toxicity tests can successfully be performed in artificial soils containing bentonite clay, and that the clay type used can significantly influence the toxicity of carbendazim and imidacloprid to earthworms, with toxicity generally being the highest in soils prepared with kaolin clay compared to bentonite clay. For both pesticides, the lower toxicity in bentonite soil appears to be caused by the higher CEC and larger distance between clay sheets in bentonite than in kaolinite clay, allowing the molecules to position themselves in between the clay sheets, and thereby reducing their bioavailability and toxicity. Overall, these findings suggest that kaolin is a suitable clay type for standardized artificial soil, as it exhibited the highest toxicity, and thus provided a “worst-case” scenario.

## Supplementary information

Supplementary_Information_Revised

## Data Availability

The raw data from the toxicity tests have been deposited on Zenodo and can be accessed on 10.5281/zenodo.13886310.
