# Supplementary material for: The effect of clay type on the toxicity of carbendazim and imidacloprid to the earthworm Eisenia andrei in artificial soils
Source: Ecotoxicology. 2025 Apr 26;34(6):925–34. doi: 10.1007/s10646-025-02889-6 (PMC12325473; doi:10.1007/s10646-025-02889-6)
Supplement: Supplementary file 1 — Supplementary_Information_Revised [file 10646_2025_2889_MOESM1_ESM.docx]

# Supplementary information to:

**The Effect of Clay Type on the Toxicity of Carbendazim and Imidacloprid to the Earthworm *Eisenia andrei* in Artificial Soils**

Bart G. van Hall^1^, Shuan Meijer^1^, Anna C. Pelser^1^ & Cornelis A. M. van Gestel^1^

^1^ Amsterdam Institute for Life and Environment (A-LIFE), Faculty of Science, Vrije Universiteit Amsterdam, De Boelelaan 1108, 1081 HZ Amsterdam, The Netherlands

**Figure S1. pH-calibration curves used to determine the amount of CaCO_3_ needed to set the pH (0.01M CaCl_2_) of artificial soils constructed with kaolin and bentonite clay to 6.0 ± 0.5.** To construct the curves, 5.0 ± 0.1 g of dry artificial soil was weighed into individual bottles and small amounts of CaCO_3_ (0.02 – 0.07%) were added, with each treatment prepared in duplicate. The soils were shaken with 25 mL 0.01 M CaCl_2_ for two hours at 200 rpm. The pH of the suspensions was measured after settling overnight.

**Table S1. Average (± SD) soil pH (0.01 M CaCl_2_) in the (solvent) control (n = 2 for imidacloprid, n = 4 for carbendazim) and highest test concentrations (n = 2) at the start (t = 0) and end (t = 56) of earthworm (*Eisenia andrei*) toxicity tests in artificial soils with different clay types.**

| **Pesticide** | **Clay type** | **(Solvent) control** | | **Highest test concentration** | |
| --- | --- | --- | --- | --- | --- |
|  |  | **t = 0** | **t = 56** | **t = 0** | **t = 56** |
| **Imidacloprid** | **Kaolin** | 5.49 ± 0.03 | 5.34 ± 0.01 | 5.54 ± 0.00 | 5.19 ± 0.03 |
|  | **Bentonite** | 5.78 ± 0.01 | 5.72 ± 0.03 | 5.73 ± 0.01 | 5.97 ± 0.05 |
|  |  |  |  |  |  |
| **Carbendazim** | **Kaolin** | 5.37 ± 0.03 | 5.45 ± 0.02 | 5.46 ± 0.03 | 5.76 ± 0.04 |
|  | **Bentonite** | 5.77 ± 0.04 | 5.80 ± 0.03 | 5.72 ± 0.03 | 6.02 ± 0.02 |


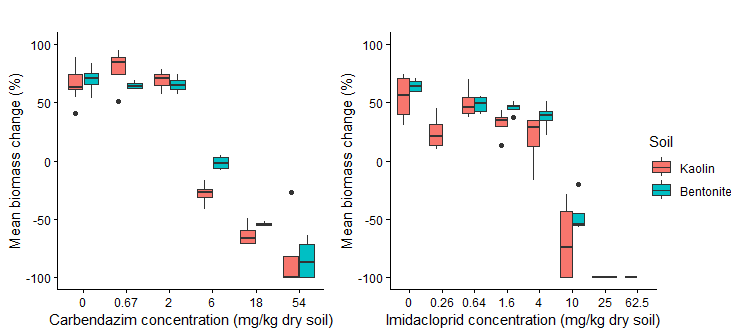
 **Figure S2. Mean biomass change (%) of earthworms *Eisenia andrei* after four weeks of exposure to different concentrations of carbendazim (left) and imidacloprid (right) in artificial soils prepared with kaolin and bentonite clay.**
